# Double-stranded RNA induces chicken T-cell lymphoma apoptosis by TRIF and NF-κB

**DOI:** 10.1038/s41598-017-07919-w

**Published:** 2017-08-08

**Authors:** Haitao Zou, Ruixue Su, Jing Ruan, Hongxia Shao, Kun Qian, Jianqiang Ye, Yongxiu Yao, Venugopal Nair, Aijian Qin

**Affiliations:** 1 Ministry of Education Key Lab for Avian Preventive Medicine, Yangzhou University, No. 12 East Wenhui Road, Yangzhou, Jiangsu 225009 P. R. China; 2 Jiangsu Co-innovation Center for Prevention and Control of Important Animal Infectious Diseases and Zoonoses, No. 12 East Wenhui Road, Yangzhou, Jiangsu 225009 P. R. China; 3 Jiangsu Key Lab of Zoonosis, No. 12 East Wenhui Road, Yangzhou, Jiangsu 225009 P. R. China; 4 The Pirbright Institute, Ash road, Pirbright, Working, Surrey, GU24 0NF United Kingdom; 5 UK-China Centre of Excellence for Research on Avian Diseases, 169 Huanghe 2nd Road, Binzhou, Shandong P. R. China

## Abstract

Toll-like receptor-3 (TLR3), a member of the pathogen recognition receptor family, has been reported to activate immune response and to exhibit pro-apoptotic activity against some tumor cells. However it is unclear whether TLR3 has same function against chicken lymphoma. In this paper we investigated the effect of TLR3 activation on a Marek’s disease lymphoma-derived chicken cell line, MDCC-MSB1. The TLR3 agonist poly (I:C) activated TLR3 pathway and inhibited tumor cells proliferation through caspase-dependent apoptosis. Using pharmacological approaches, we found that an interferon-independent mechanism involving Toll-IL-1-receptor domain-containing adapter-inducing IFN-α (TRIF) and nuclear factor κB (NF-κB) causes the apoptosis of MDCC-MSB1 cells. This is the first report about the function of TLR3 in chicken T-cell lymphoma, especially in signal pathway. The mechanisms underlying TLR3-mediated apoptosis may contribute to the development of new drug to treat lymphomas and oncovirus infections.

## Introduction

Double-stranded RNA (dsRNA) is a typical pathogen-associated molecular pattern (PAMP), representing either genomic or life cycle intermediate material of many viruses. It is recognized by Toll-like receptor 3 (TLR3), double-stranded RNA-activated protein kinase (PKR), retinoic acid-inducible gene I protein (RIG-I) and melanoma differentiation-associated protein 5 (MDA5), resulting in a strong antiviral response through type I interferon (IFN) response^1^. Moreover, the dsRNA analog poly (I:C) induces apoptosis in different cell types, apparently through multiple pathways^2–5^. In particular, poly (I:C) leads to the apoptosis of several tumor cell types, including head and neck cancer, lung cancer, prostate cancer, and breast cancer, suggesting a significant role for the TLR3 pathway in immune response against tumor^6–10^.

dsRNA binding leads to TLR3 dimerization and activation of its Toll-IL-1-receptor (TIR) cytoplasmic domain, which recruits the adapter molecule TIR domain-containing adapter inducing IFN-α (TRIF). TRIF continues to recruit tumor necrosis factor (TNF) receptor-associated factor 6 (TRAF6) and the receptor interacting protein 1 (RIP1) serine–threonine kinase to activate NF-κB, or TRAF3 for the activation of IFN regulatory factor 3 (IRF3) and the type I IFN response^11^. Both NF-κB and IRF3 are involved in cell survival and apoptosis^6, 12^. In addition, some reports indicated that other protein involved in TLR3 pathway such as RIP1, TRIF and TRAF6 can directly or indirectly regulate apoptosis^13–16^.

Marek’s disease, which is caused by Marek’s disease virus (MDV), presents with typical T-cell lymphomas clinical symptom and solid visceral tumors that contain transformed CD4^+^ T cells^17^. MDV-chicken is a well-defined small-animal model for understanding some of the principles of human disease, in particular, general tumorigenesis, and virus-induced lymphomagenesis^18^. Although activation of TLR3 pathway has been reported to cause apoptosis of various tumor cells, no evidence indicates whether it is effective on lymphomas. TLR3 function was found to be repressed when the MDV infection enters the tumor transformation phase^19, 20^. Additionally, poly (I:C) inhibited lymphomas development in chicken, suggesting a potential powerful mechanism from TLR3 activation that targets lymphoma^21^. However it is still unclear how the TLR3 pathway achieves this function. In this study, we investigated the effects of poly (I:C) on Marek’s disease lymphoma-derived chicken cell line and explored the TLR3-dependent signaling pathways that drive apoptosis in lymphomas cells.

## Results

### Poly (I:C) directly induces apoptosis in MDCC-MSB1 cell

**Figure 1:**
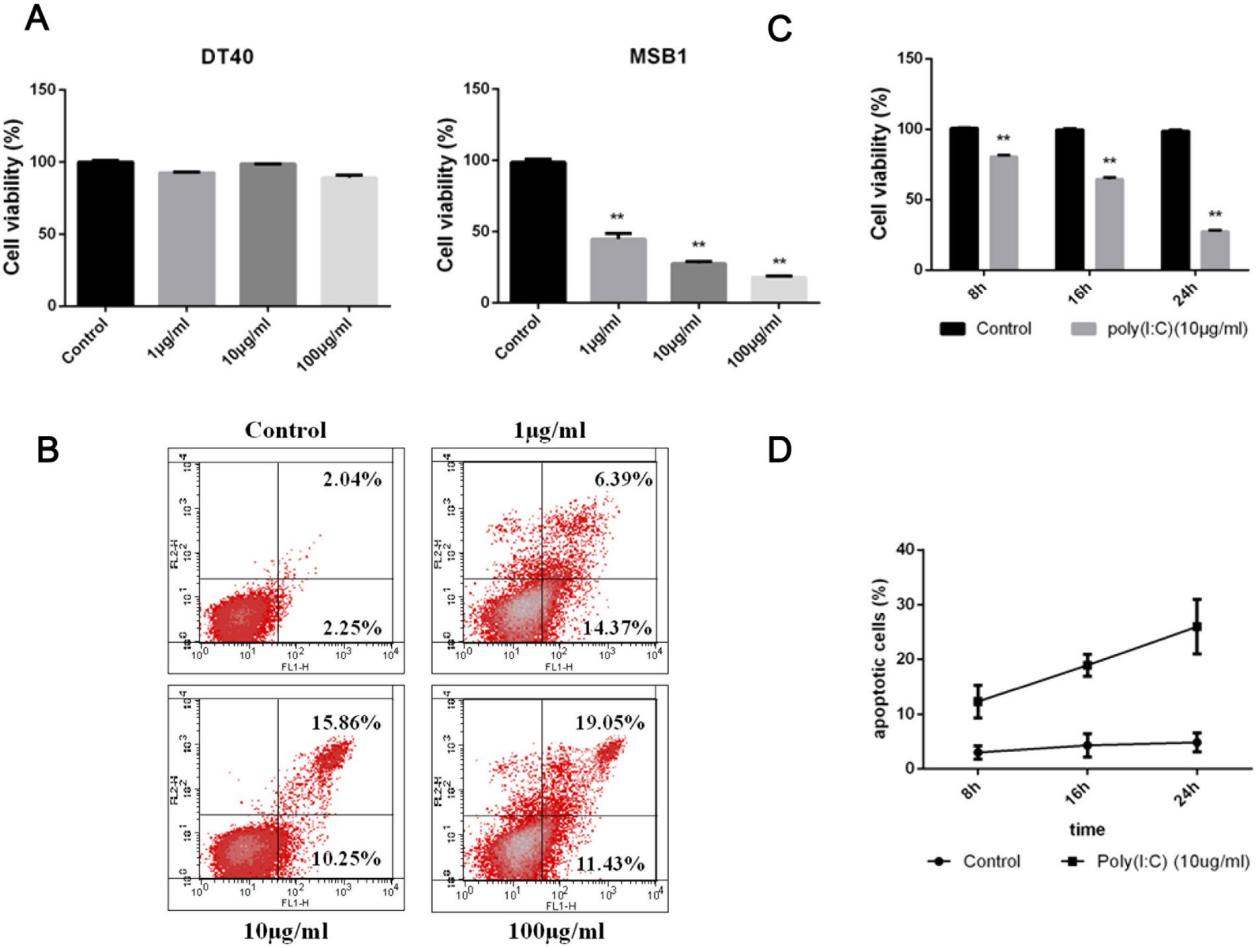
Apoptosis of chicken lymphoma cells is induced by the synthetic dsRNA analogue poly (I:C). (**A**) Chicken lymphoma cells were cultured with or without increasing doses of poly (I:C) (1 μg/ml, 10 μg/ml, 100 μg/ml) poly (I:C) for 24 h and viability is expressed as a percentage. (**B**) MDCC-MSB1 cells were cultured for 24 h with or without increasing doses of poly (I:C) (1 μg/ml, 10 μg/ml, 100 μg/ml), and apoptosis was detected by annexin V and PI staining. (**C**) MDCC-MSB1 cells were cultured with or without poly (I:C) (10 μg/ml), and the percentage of cell viability was measured at the indicated time points. (**D**) MDCC-MSB1 cells were cultured with or without poly (I:C) (10 μg/ml) and the percentage of apoptotic cells expressed as a percentage at the indicated time points. The *asterisk* (*) or *double asterisk* (**) respectively indicates p < 0.05 or p < 0.01 in statistical difference from controls. The bars represent an average of multiple experiments.

To investigate the effect of TLR3 agonist on chicken lymphoma, the Marek’s disease lymphoma-derived chicken cell line MDCC-MSB1 cells and the avian leukosis virus (ALV) lymphoma-derived chicken cell line DT40 cells were cultured with 1 μg/ml, 10 μg/ml or 100 μg/ml dsRNA analog poly (I:C) for 24 h. All three groups of MDCC-MSB1 cells showed a significant decrease in cell viability as measured by a CCK-8 assay, with a dosage of 100 μg/ml exhibiting the most dramatic decrease (Fig. 1A). No significant change of cell viability was found in DT40 cells stimulated with poly(I:C). The decrease in cell viability due to apoptosis was further illustrated by annexin V and PI staining. Poly (I:C) induced significant dose-dependent apoptosis in MDCC-MSB1 cell line, with an apoptotic percentage range from 20.76 to 30.48% (Fig. 1B). At the same time, apoptosis at different times was also measured by CCK-8 assay and annexin V staining (Fig. 1C,D). All of the results demonstrated that poly(I:C) directly induced the apoptosis of chicken T-cell lymphoma in a dose-dependent manner.

### Poly (I:C) triggered activation of intrinsic and extrinsic caspase cascades

**Figure 2:**
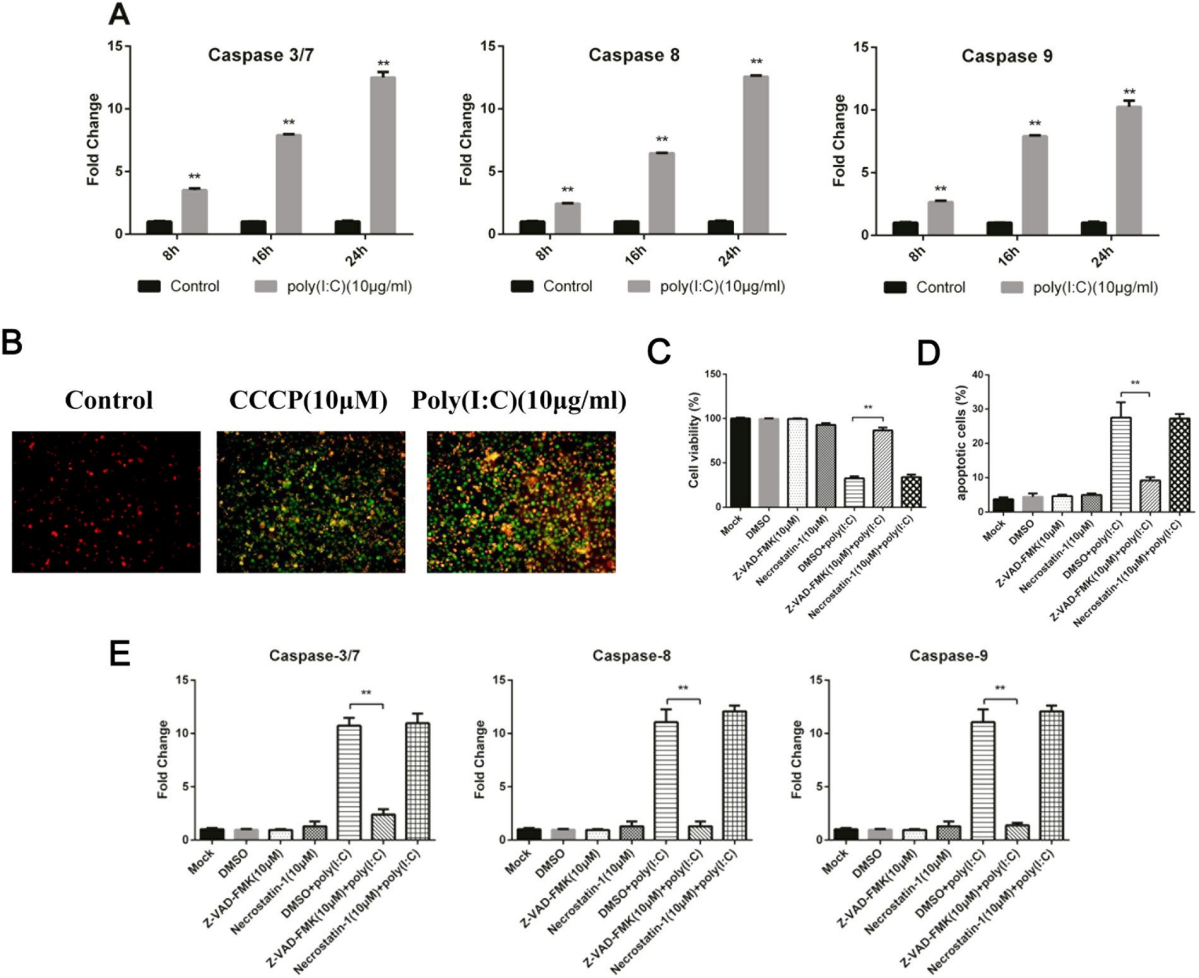
The activation of caspase pathway in MDCC-MSB1 cells cultured with poly (I:C). (**A**) The activities of caspases 3/7, 8, and 9 were measured in MDCC-MSB1 cells cultured with poly (I:C) (10 μg/ml) for 8, 16 and 24 h.The relative activity of the caspases was calculated as in Materials and Methods. (**B**) MDCC-MSB1 cells were cultured for 24 h with or without poly (I:C) (10 μg/ml), then stained with 5 μM JC-1 dye for 10 min at 37 °C to evaluate mitochondrial membrane potential integrity. Mitochondria with normal membrane potentialare shown in red, and mitochondria experiencing a loss of membrane potential are indicated in green. (**C**) MDCC-MSB1 cells were pre-treated with pan-caspase inhibitor Z-VAD-FMK, Necrostatin-1 or DMSO (used as control) before culture with or without poly (I:C) (10 μg/ml) for 24 hours. The percentage of cell viability is shown. (**D**) MDCC-MSB1 cells were pre-treated with the pan-caspase inhibitor Z-VAD-FMK, Necrostatin-1 or DMSO (used as control) before culture with or without poly (I:C) (10 μg/ml) for 24 hours. Results are expressed as a percentage of the apoptotic cells. (**E**) The caspase activity of MDCC-MSB1 cells, obtained as described in (**A**), was measured. The *asterisk* (*) or *double asterisk* (**) respectively indicates p < 0.05 or p < 0.01 in statistical difference from controls. The bars represent an average of multiple experiments.

The function of caspases in apoptosis induced by TLR3 agonist was accessed. Caspase activity was measured in the culture medium as well as in cells treated with poly (I:C) using relative light units (RLU) of luminescence at 8, 16, and 24 h. The activity of caspases 3/7, 8, and 9 was significantly enhanced from 8 h to 24 h in poly (I:C) treated cells compared to untreated cells (Fig. 2A). Then, MDCC-MSB1 cells were pre-treated with 10 μM of the pan-caspase inhibitor Z-VAD-FMK for 2 hours followed culture with poly (I:C) for 24 h. The effect of Z-VAD-FMK was measured by cell viability, annexin V staining, and caspase activity (Fig. 2C–E). The activity of caspases 3/7, 8, and 9 was inhibited completely in the Z-VAD-FMK-treated group. Moreover, Z-VAD-FMK-treated group was equivalent to the control group, indicating that the death of MDCC-MSB1 cells occurred entirely through a caspase-dependent mechanism. We also found that the necrostatin-1 had no effect on MDCC-MSB1 cells death induced by poly (I:C). It suggested that the MDCC-MSB1 cell death induced by poly (I:C) was apoptosis rather than necroptosis. The activation of caspases 8 and 9 represents extrinsic and intrinsic caspase cascades. In other words, both extrinsic and intrinsic caspase cascade participated in the apoptosis process. At the same time, we investigated mitochondria membrane potential (MMP) of MDCC-MSB1 cells treated with poly (I:C) by JC-1 dye staining. At 24 hours, MMP was almost completely down-regulated in cells treated with poly (I:C) (Fig. 2B). This result confirmed the involvement of the intrinsic caspase cascade in the apoptosis process.

### Pro-apoptotic molecules and adaptors involved in TLR3 pathway were up-regulated during the apoptosis

**Figure 3:**
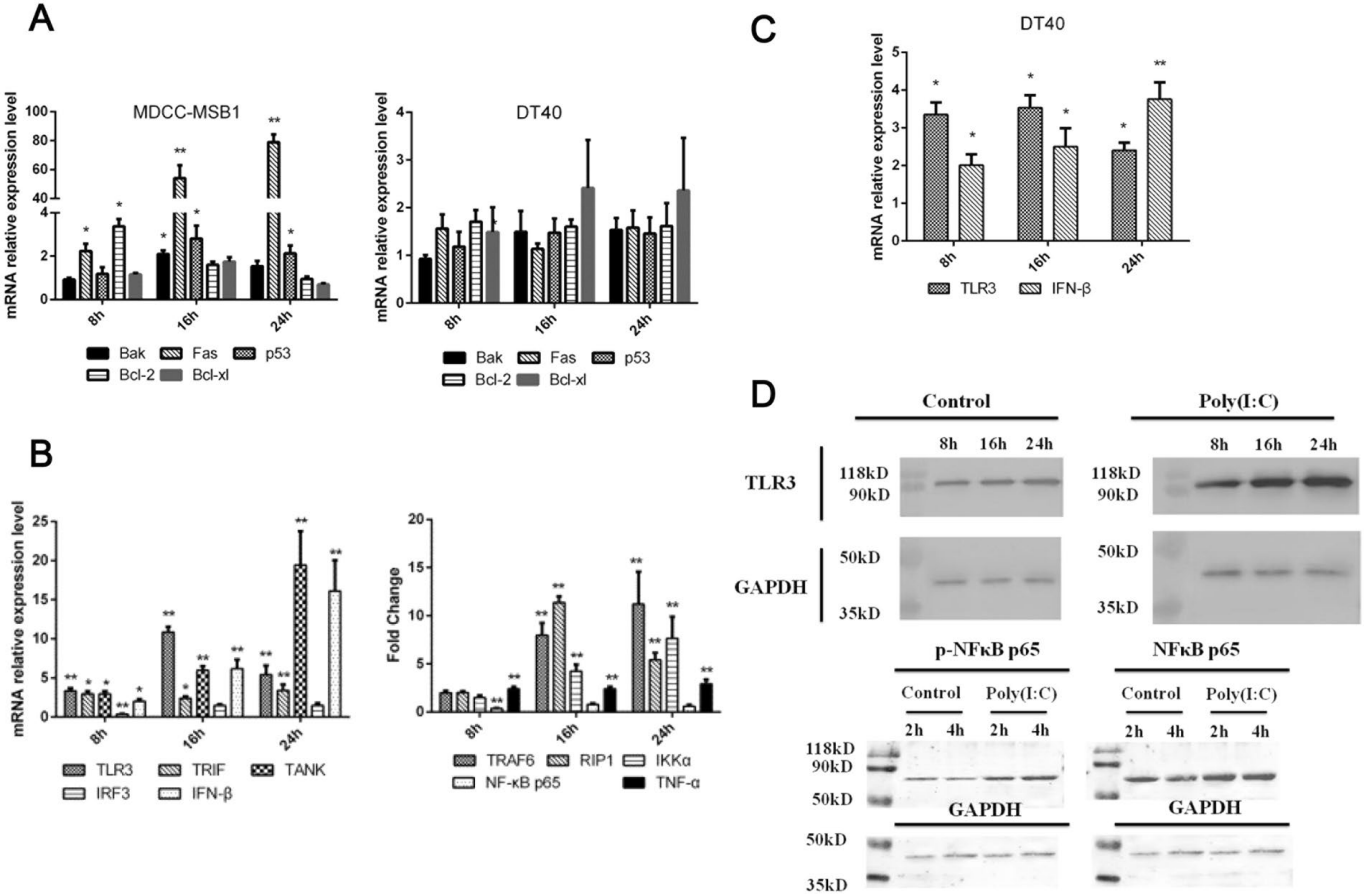
Transcriptional profiling of pro-apoptotic factors and molecules involved in TLR3 pathway in MDCC-MSB1 cells cultured with poly (I:C). (**A**) Transcriptional kinetic of factors associated with apoptosis in MDCC-MSB1 cells and DT40 cells cultured with poly (I:C) (10 μg/ml) at 8, 16, or 24 h. (**B**) Transcriptional kinetic of molecules involved in TLR3 pathway in MDCC-MSB1 cells cultured with poly (I:C) (10 μg/ml) at 8, 16, or 24 h. (**C**) Transcriptional kinetic of TLR3 and IFN-β in DT40 cells cultured with poly (I:C) (10 μg/ml) at 8, 16, or 24 h. (**D**) Expression of TLR3 and phosphorylated NF-κB p65 in MDCC-MSB1 cells cultured with poly (I:C) (10 μg/ml) at 8, 16, or 24 h. The *asterisk* (*) or *double asterisk* (**) respectively indicates p < 0.05 or p < 0.01 in statistical difference from controls. The bars represent an average of multiple experiments.

To investigate the possible molecular mechanism of poly (I:C)-triggered MDCC-MSB1 cell apoptosis, the expression of several factors associated with apoptosis were detected by real-time PCR (Fig. 3A). At 8 h, *Fas* and *Bcl-2* were significantly up-regulated, whereas *Bak* and *p53* remained unchanged. Besides significant increase of *Bak* and *p53*, a dramatic up-regulation of *Fas* transcript was found at 16 and 24 h. No significant change of *Bcl-2* and *Bcl-xl* was found at 16 and 24 h. Compared with MDCC-MSB1 cells, no significant up-regulation of anti-apoptotic and pro-apoptotic expression was found in DT40 cells stimulated with poly(I:C). Taken together, the up-regulation of pro-apoptotic factors further confirmed that poly(I:C) induces the apoptosis of MDCC-MSB1 cells via both intrinsic and extrinsic apoptosis pathway.

At the same time, a series of adaptors and down-stream factors involved in the TLR3 pathway were assessed by real-time PCR to further characterize TLR3 activation (Fig. 3B). At 8 h, the molecules associated with production of type I IFN were significantly up-regulated except for *IRF3*, whereas the adaptors related to *NF-κB* activation remained unchanged. Without *IRF3* and *NF-κB p65*, almost all of these adaptors were significantly up-regulated at 16 h and 24 h. As typical downstream effectors, *IFN-β* and *TNF-α* obviously increased at each time point, but their function during apoptosis were undefined. Similarly, *TLR3* and *IFN-β* were also up-regulated in DT40 cells stimulated with poly(I:C), but we have not found any apoptosis in DT40 cells (Fig. 3C). According to the results of western-blot analysis, up-regulation of TLR3 and phosphorylation of NF-κB p65 were confirmed, which is the activation of TLR3 pathway (Fig. 3D).

### TRIF is involved in MDCC-MSB1 cell apoptosis induced by poly (I:C), independently of PKR

**Figure 4:**
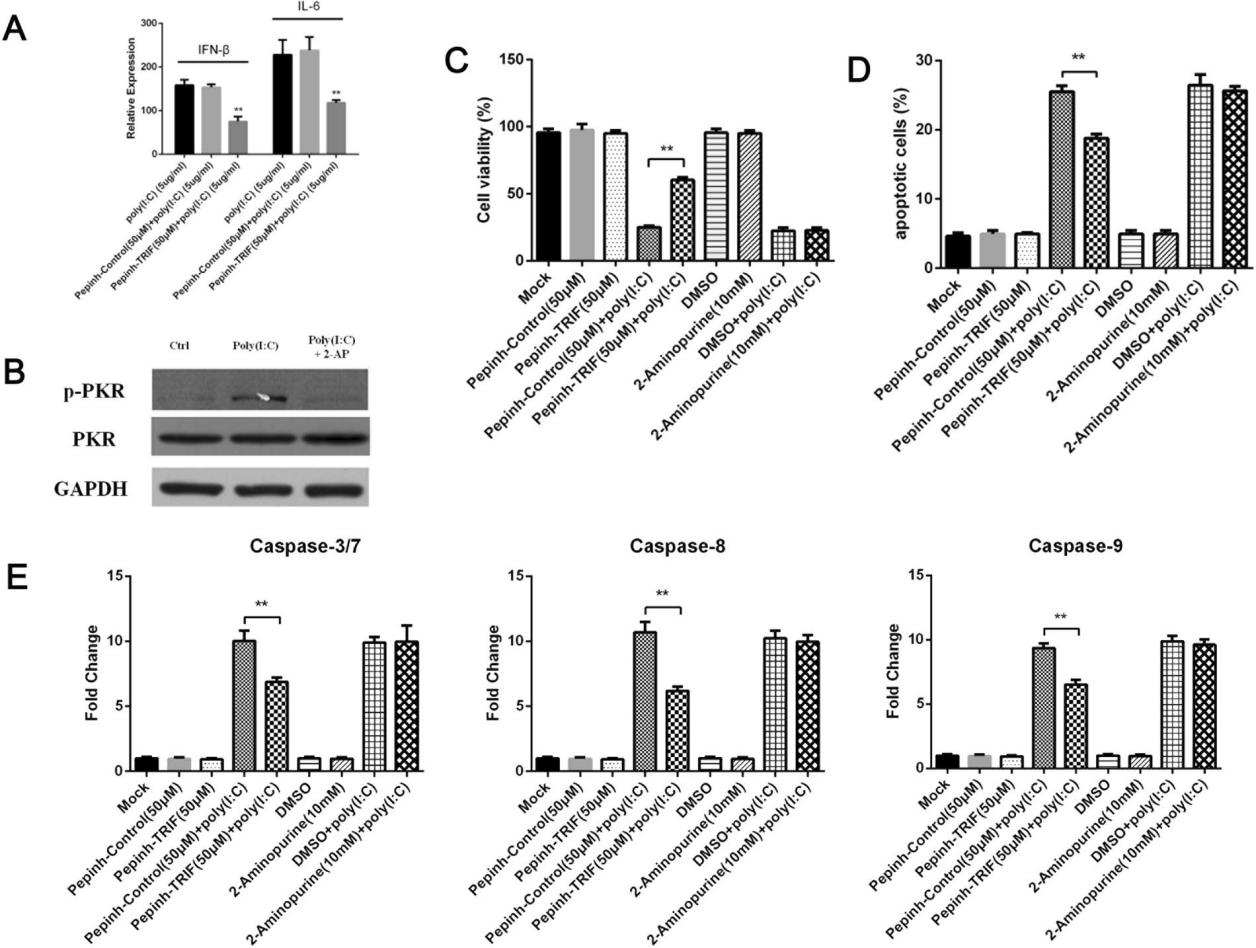
Poly (I:C)-induced apoptosis is mediated by TRIF independent of PKR. (**A**) Verification of TRIF inhibitor effect in avian cells. (**B**) Verification of PKR inhibitor effect in avian cells. (**C**) MDCC-MSB1 cells were pre-treated with the Pepinh-Control (used as control), Pepinh-TRIF, 2-aminopurine, or PBS (used as control) before culture with or without poly (I:C) (10 μg/ml) for 24 hours. The percentage of cell viability is shown. (**D**) The percentage of apoptotic cells among MDCC-MSB1 cells, treated as described in C, was shown. (**E**) The caspase activity of MDCC-MSB1 cells, obtained as described in D, was measured. The *asterisk* (*) or *double asterisk* (**) respectively indicates p < 0.05 or p < 0.01 in statistical difference from controls. The bars represent an average of multiple experiments.

TRIF is the critical adaptor protein that connects TLR3 and downstream signaling cascade. The inhibitors Pepinh-TRIF and 2-Aminopurine were used to efficiently suppress TRIF and PKR activation, respectively, to determine whether PKR or TLR3 was involved in dsRNA-induced MDCC-MSB1 cell apoptosis. The suppression of TRIF with the specific inhibitor effectively attenuated poly (I:C)-induced apoptosis, whereas apoptosis occurred normally after the inactivation of PKR (Fig. 4C,D). At the same time, the inhibitor Pepinh-TRIF significantly decrease the activity of caspases 3/7, 8, and 9, suggesting a role for TRIF in initiation of caspase activation (Fig. 4E). Although the highest work concentration of Pepinh-TRIF was used in this work according to the manufacturer’s instructions, it can only efficiently inhibit rather than completely prevent poly (I:C)-induced apoptosis and caspase activity, which can be ascribe to the lower inhibitory effect in avian cells (an inhibition of 50–60%, Fig. 4A) than in mammal cells. Taken together, these data demonstrate that synthetic dsRNA induces the apoptosis of MDCC-MSB1 cells in a TRIF-dependent manner without PKR involvement.

### TLR3-induced apoptosis is mediated by NF-κB activation independent of type I IFN signaling

**Figure 5:**
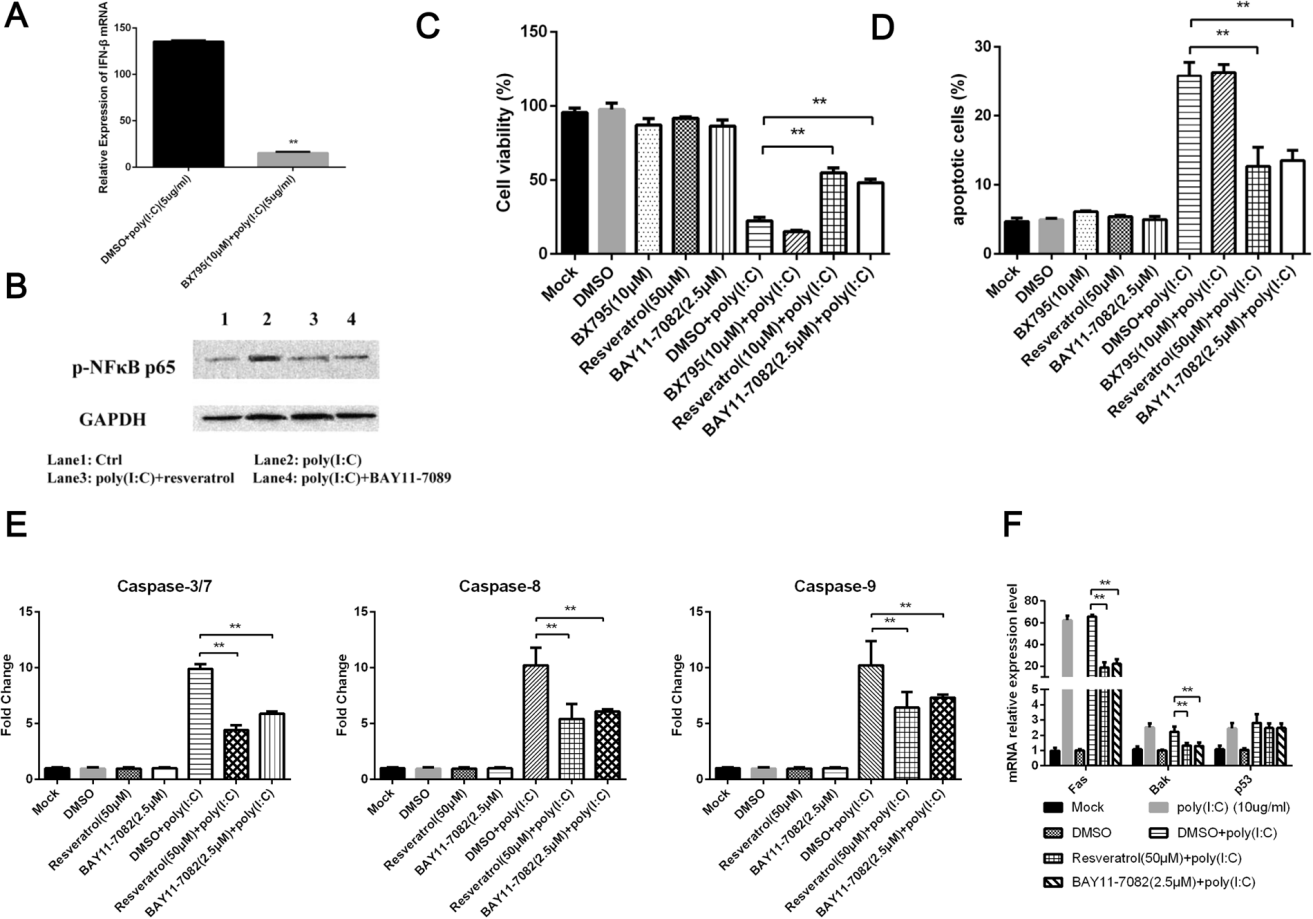
Poly(I:C)-induced apoptosis mediated by NF-κB activation, independently of type I IFN signaling. (**A**) Verification of IRF3 inhibitor effect in avian cells. (**B**) Verification of NF-κB inhibitor effect in avian cells. (**C**) MDCC-MSB1 cells were pre-treated with the BX795, resveratrol, BAY11-7089, or DMSO (used as control) before culture with or without poly (I:C) (10 μg/ml) for 24 hours. The percentage of cell viability is shown. (**D**) The percentage of apoptotic cells in MDCC-MSB1 cells, treated as described in C, was shown. (**E**) The caspase activity of MDCC-MSB1 cells, obtained as described in D, was measured. (**F**) Transcriptional kinetic of pro-apoptotic factors in MDCC-MSB1 cells, treated as described in C, was shown. The *asterisk* (*) or *double asterisk* (**) respectively indicates p < 0.05 or p < 0.01 in statistical difference from controls. The bars represent an average of multiple experiments.

TLR3 has been known to mediate type I IFN response and NF-κB activation through TRIF. To determine whether a type I IFN response or NF-κB activation was involved in dsRNA-induced apoptosis of MDCC-MSB1 cells, the activation of each pathway was efficiently suppressed using specific inhibitors BX795, resveratrol and BAY11-7082. According to the results, the suppression of NF-κB activation prevented poly (I:C)-triggered and TLR3-mediated apoptosis and caspase activation, whereas inhibition of type I IFN response was ineffective at decreasing cell death (Fig. 5C–E). Moreover, the suppression of NF-κB activation significantly decreased the transcription of *Fas* and *Bak* (Fig. 5F). It revealed that NF-κB activation regulated dsRNA-induced MDCC-MSB1 cell apoptosis through *Fas* and *Bak*. Collectively, the possible contribution of NF-κB activation to the poly(I:C)-induce cell apoptosis is to regulate expression of molecules involved in cell death and survival rather than pro-inflammatory cytokines.

## Discussion

We elucidate how the dsRNA analog poly (I:C) leads to apoptosis of MDCC-MSB1 cells. The lymphoma cells after poly (I:C) treatment were characterized by rapid apoptosis that required a caspase cascade activated by TRIF and NF-κB. Although it has been reported that poly (I:C) can induce apoptosis in several tumor cell types including head and neck cancer, lung cancer, prostate cancer, breast cancer and others^3, 6, 8–10, 22, 23^. Moreover, Kalai *et al*. first reported that dsRNA was able to induce apoptosis of human T lymphoma cell line Jurkat^24^. In their report, poly (I:C)-treatment initiated different death signaling pathways, leading to either necrotic or apoptotic cell death. Kalai *et al*. have found that caspase-8 and Fas-associated with death domain (FADD) were required and not “dispensable” for Jurkat cell apoptosis. However they did not explain how poly (I:C) triggered the activation of cell apoptosis, especially, whether TLR3 or related molecules was associated with this process. In our case, we first demonstrate the contribution of poly (I:C) to chicken T-cell lymphoma apoptosis rather than necrotic, which is different from Jurkat cells. And we explored the death signaling pathways involved in poly (I:C)-induced apoptosis of chicken lymphoma cells.

According to previous research, the mechanisms by which the activation of TLR3 triggers apoptosis are diverse in different cells. A type I IFN response induced by TLR3 pathway can be involved in tumor cells death directly or indirectly. IRF3 is thought to play an essential role in the TLR3-mediated apoptosis of prostate cancer cells and ovarian cancer cells by activating intrinsic and extrinsic apoptotic pathways^3, 23, 25^. However, poly (I:C)-induced MDCC-MSB1 cell apoptosis occurs independently of above pathway. Although the transcription of *IFN-β* mRNA was dramatically increased, the signs of apoptosis were still observed after cells were treated with specific inhibitor to block IRF3 activation and IFN-β production. Furthermore, there are several MDV-encoded miRNAs and proteins expressed in MDCC-MSB1 cells, which behave quite differently with other tumors^26, 27^. Many types of viral proteins have been identified as inhibitors of STAT1, an indispensable nucleus transcription factor that promotes transcription of interferon-stimulated genes (ISGs)^28–31^ and also plays an important role in type I IFN-induced apoptosis^32–34^. It is unclear whether MDV-encoded proteins have a similar capacity to interfere with the type I IFN pathway participating in apoptosis. In short, the production of IFN-β induced by TLR3 activation did not contribute to poly (I:C)-triggered apoptosis of MDCC-MSB1 cells.

Instead, poly (I:C)-induced MDCC-MSB1 cell apoptosis required the activation of NF-κB. In the presence of specific inhibitors to block activation of NF-κB, the percentage of apoptotic cells was significantly decreased, and caspase activity was also obviously down-regulated. NF-κB function in the regulation of cell death is quite complex^35^. In most cases, a role for NF-κB in resistance to cell death is initially apparent in several cell types^6, 36–41^. Downstream cytokines of NF-κB, such as IL-1β and IL-6 can protect cells to apoptosis^42, 43^. In this case, NF-κB plays a pro-apoptotic role in poly (I:C)-triggered apoptosis of MDCC-MSB1 cell. For example, an autocrine effect of TNF-α induced by NF-κB activation has previously been found during the apoptotic activation of human alveolar macrophages^44^. p53 directly interacts with Bak, liberating Bak from inhibitory complexes^45^. The crucial event for initiating the intrinsic mitochondria-mediated pathway of apoptosis is mitochondrial outer membrane permeabilization (MOMP) that requires the activation of Bak^46^. p53 can mediate apoptosis through inducing mitochondrial release of pro-apoptotic factors, and NF-κB is necessary for this event^47^. Other data have also shown that NF-κB activation render cells more sensitive to certain pro-apoptotic stimuli^48–52^. Furthermore, activation of NF-κB crucially contributes to apoptosis of T-cell lymphoma via Fas-dependent pathway, suggesting the pro-apoptotic of NF-κB in T-cell lymphoma apoptosis^53, 54^. Fas is a member of the death receptor family, recruiting FADD and caspase 8 to the death-inducing signaling complex to extrinsic apoptosis pathway^55, 56^. We detected high expression of the Fas and pro-apoptotic factors regulated by p53 during MDCC-MSB1 cell apoptosis. Moreover, the suppression of NF-κB activation significantly decreased the transcription of Fas and Bak. All the results were consistent with conclusions above mentioned showing that NF-κB plays a pro-apoptotic role in poly (I:C)-triggered apoptosis of MDCC-MSB1 cells.

During the process of apoptosis, caspase 3, caspase 8 and caspase 9 were activated and general caspase inhibitor completely abolished the activity of caspase to trigger MDCC-MSB1 cell apoptosis. This suggests that the caspase apoptotic pathway acts as an effector to perform apoptosis after poly(I:C) stimulation. In fact, a number of mechanisms to bridge TLR3 pathway and caspase cascade have been described to date. For example, TLR3 activation induces TAp63α, a p53-related protein, which contributes to the activation of caspase 8 and caspase 9 via death receptors and mitochondria in human umbilical vein endothelial cells^57^. Activation of TLR3 by dsRNA recruits caspase 8 to form a death-signalling complex in the presence of RIP1, which appears not to be stringently dependent on Fas-associated with death domain (FADD) and results in initiation of the extrinsic apoptosis pathway in lung cancer cells^58^. RIP1 is necessary for the recruitment of caspase 8 to TLR3. cIAPs-mediated ubiquitination of RIP can negatively regulate TLR3 to caspase 8 death complex formation and apoptosis. This report not only showed the direct activation of caspase-8 by TLR3 without FADD but also reflected the important role of RIP1 in TLR3-mediated apoptosis. Considering the inhibition of type I IFN and cytokines induced by TLR3, perhaps the adaptors involved in the TLR3 pathway are vital for activating the caspase cascade to induce apoptosis in our case.

Adaptors involved in the TLR3 pathway also exhibit the capacity to regulate proliferation and apoptosis. RIP1 is one of the core adaptors in the apoptotic pathway that induces NF-κB activation to control the expression of proteins associated with cell death and survival^59^. We detected significantly high expression of the *RIP1* transcript during MDCC-MSB1 cell apoptosis, strongly suggesting that RIP1 contributes to apoptosis. In addition to RIP1, as mentioned above, TRAF6 directly interacts with the death effect domain of pro-caspase 8 through the C-terminal TRAF domain and activates caspase 8 through the N-terminal RING domain in humans^60^. TRIF has been proven to be a key molecule for apoptosis regulation. Apoptosis triggered by TRIF is depend on its receptor-interacting protein homotypic interaction motif^61^. And TRIF is critical for TLR3-induced activation of NF-κB^11^. In our case, TRIF inhibitor could significantly suppress MDCC-MSB1 cell apoptosis induced by poly(I:C). TRIF may directly trigger cell apoptosis. On the other hand, TRIF acting NF-κB was critical for MDCC-MSB1 cell apoptosis induced by ply(I:C). Although TRIF is a core adaptor in TLR3 pathway, it is also regarded as a critical component of other RNA sensors pathway. Zhang, Z. *et al*. reported that DDX1, DDX21 and DHX36 helicases formed a complex with the adaptor molecule TRIF to sense dsRNA in dendritic cells^62^. It should be investigated whether other RNA helicases can trigger or regulate cell apoptosis in chicken T cells in future.

Although the original goal was to trigger an innate immune response against chicken T-cell lymphoma, the above data suggest that TLR3 agonists might have a direct pro-apoptotic effect on tumour cells. However, not every lymphoma cell line we tested showed apoptotic induction after poly (I:C) treatment. For example, the avian leukosis virus (ALV) lymphoma-derived chicken cell line DT40 was tested, and no simple correlation was apparent between TLR3 expression in the resting state and poly (I:C)-induced apoptosis. Moreover, TLR3 activation is also unable to cause normal CD4^+^ T cell death. Why does TLR3 activation lead to such different outcome? Ripoptosome is a large cell death-inducing platform, which consists of core components RIP1, FADD and caspase 8 independently of TNF, CD95L/FasL, TRAIL, death-receptors, and mitochondrial pathways^63^. Ripoptosome formation was found with a capacity of converting pro-inflammatory cytokines into prodeath signals. cFLIP isoforms differentially regulate TLR3-mediated cell death by regulation of the ripoptosome^64^. In other words, ripoptosome maybe a switch controlled the convert of TLR3 function between induction of pro-inflammatory and pro-apoptosis. Whether there is the formation of ripoptosome during poly (I:C)-induced MDCC-MSB1 cell apoptosis, it might be an important unanswered question that should be focused on in subsequent work. To conclude, these data inspire the application of poly (I:C) as antiviral or anti-tumour agents against certain types of lymphoma or oncoviral infections because they can directly kill the tumour and enhance the host’s immune response against it.

## Methods

### Cells and reagents

The MDCC-MSB1 cell line, a Marek’s disease lymphoma-derived chicken cell line, was cultured at 38.5 °C in 5% CO_2_ in RPMI 1640 medium (Gibco, USA) containing 10% fetal calf serum (Gibco, Australia) and 10% tryptose phosphate broth (Sigma-Aldrich, USA). Avian leukosis virus (ALV) lymphoma-derived chicken cell line DT40 was cultured at 38.5 °C in 5% CO_2_ in RPMI 1640 medium (Gibco, USA) containing 10% fetal calf serum (Gibco, Australia), 5% chicken serum (Gibco, USA) and 5% tryptose phosphate broth (Sigma-Aldrich, USA). Poly (I:C) was obtained from Sigma-Aldrich (USA). The pan-caspase inhibitor Z-VAD-FMK was purchase from Beyotime Biotechnology (Nantong, China). The inhibitors Pepinh-TRIF, resveratrol, BX795, and 2-aminopurine were purchase from InvivoGen (USA). Necrostatin-1 and BAY11-7082 was purchased from Abcam (USA).

### Cell viability analysis

CCK-8 cell survival assays were performed according to the manufacturer’s instructions (Beyotime Biotechnology, Nantong, China). Briefly, cells were seeded at 5 × 10^4^ cells/well in 96-well plates, and after 8, 16, or 24 h culture with or without poly (I:C), CCK-8 reagent was added to culture medium. After incubate at 37 °C for 4 h, the absorbance of each well was read at 450 nm. The results were calculated as the absorbance ratio of poly (I:C)-treated to untreated cells.

### Detection of apoptotic cells

For apoptosis measurements, harvested cells were stained with FITC Annexin V Apoptosis Detection Kit I (BD Pharmingen, USA) according to the manufacturer’s instructions. Harvested cells were washed with cold PBS twice and resuspended in binding buffer containing annexin V-FITC and PI. Cell populations labeled with FITC and PI were analyzed by flow cytometry (CyAn ADP7, Beckman Coulter) with Summit 4.3 software (Beckman Coulter), and showed as a percentage.

### Determination of caspase activity

The activity of caspases 3/7, 8, and 9 was detected using the Caspase Glo assay kit (Promega, USA) according to the manufacturer’s instructions. Briefly, cells were seeded at a density of 5 × 10^4^ cells/wells in 96-well plates, and after 8, 16, or 24 h culture with or without poly (I:C), 100 μL Caspase Glo reagent was added to the culture medium. After mixing, the cells were transferred to a 96-well white-wall plate for incubation at room temperature in the dark for 30 min. The luminescence of the samples was measured with a luminometer (Glomax multi+, Promega). Luciferase activity was normalized against the amount of total protein. The fold change in protease activity was calculated by comparing the luciferase activity of the treated cells with that of the untreated cells.

### Determination of the mitochondrial membrane potential (MMP)

Cells treated with poly (I:C) was collected and washed twice with cold PBS. The pipetted cell was re-suspended with PBS supplemented with 5 μM JC-1 dye (Sigma-Aldrich, USA). After incubation at 37 °C for 15 min, the cells were observed with a fluorescence microscope.

### Real-time PCR

Total RNA was extracted from cells with Trizol (Invitrogen), and cDNA was synthesized using PrimeScript™ RT reagent Kit (Takara, Dalian, China). Real-time PCR was performed using SYBR Premix ExTaq II (Takara Dalian, China) in an ABI 7500 real-time PCR system (Applied Biosystems, CA, USA). The specific primers used for real-time PCR were designed according to previously published sequences and shown in Table 1. The mRNA expression level was normalized against the level of chicken GAPDH mRNA. The fold change of all genes expression was calculated by the 2^−ΔΔCt^ method.

**Table 1:** Primers used for real-time PCR.

Target Gene	Sequence	Product size (bp)	Accession number
GAPDH	F 5′-AGGGTGGTGCTAAGCGTGTTA-3′	78	NM_204305.1
	R 5′-TCTCATGGTTGACACCCATCA-3′		
TRAF6	F 5′-TCTGTTTGTCCACACGATGC-3′	145	XM_421089.2
	R 5′-TATCTCTGGCTTGGCTTCCA-3′		
TLR3	F 5′-GCACCTGTGAAAGCATTG-3′	99	NM_001011691.3
	R 5′-TAGGCGGGGTGTTACAAATG-3′		
TRIF	F 5′-CACAGACCTTGCAATCCTCA-3′	134	NM_001081506.1
	R 5′-ATCACTGGTGCTCACTTCAC-3′		
TANK	F 5′-GAGGAGTGGGCCAAGATGAG-3′	185	NM_001277871.1
	R 5′-GTCTCTGTGCTCCCGGTTAAA-3′		
IKKα	F 5′-CTTTCATCTATGGCAACTCCTG-3′	244	NM_001012904.1
	R 5′-ATGTCCAAACCAAGACGTGAT-3′		
IRF3/7	F 5′-GAGCCTCCTCCCTCAACAGT-3′	118	NM_205372.1
	R 5′-AGGGACACAGGAAGGGAGTG-3′		
NF-κB p65	F 5′-GCCAGGTTGCCATCGTGTTCC-3′	181	NM_205129.1
	R 5′-CGCGTGCGTTTGCGCTTCTC-3′		
IFN-β	F 5′-GCCCACACACTCCAAAACACTG-3′	151	NM_001024836.1
	R 5′-TTGATGCTGAGGTGAGCGTTG-3′		
RIP1	F 5‘-AGTGCTCCAAAAAGTCCCAGTACC-3′	211	XM_015275820.1
	R 5′-GGTCTCTTCTTTGGTCAGCCG-3′		
TNF-α	F 5′-TGTGTATGTGCAGCAACCCGTAGT-3′	229	NM_204267.1
	R 5′-GGCATTGCAATTTGGACAGAAGT-3′		
Bak	F 5′-ATGGATGCCTGTCTGTCCTGTTC-3′	106	NM_001030920.1
	R 5′-GCAGAGCAGTCCAAAGACACTGA-3′		
p53	F 5′-GTCCCATCCACGGAGGATTATG-3′	92	NM_205264.1
	R 5′-GAGTAAGTGCAGGTGACCGATT-3′		
Fas	F 5′-CTTCTCGGTGTGAACATTGCG-3′	219	NM_001199487.1
	R 5′-GCTGGTGGGTCAGGTCAACATC-3′		
Bcl-2	F 5′-GATGACCGAGTACCTGAACC-3′	114	NM_205339.2
	R 5′-CAGGAGAAATCGAACAAAGGC-3′		
Bcl-xl	F 5′-TCCTCATCGCCATGCTCAT-3′	69	NM_001025304.1
	R 5′-CCTTGGTCTGGAAGCAGAAGA-3′		

### Western-blot

Cells were lysed with RIPA buffer with protease inhibitors. The samples were loaded with 5 × denaturing sample buffer and separated by 12% SDS-PAGE. The proteins were transferred to 0.2 μm nitrocellulose (NC) membrane and were subsequently analysed by immunoblot with the relevant antibodies. The blots were developed by using chemiluminescence (Protein Simple, Fluorchem E FE0605). The monoclonal antibody used to detect GAPDH was from Abcam (Cat. No. ab9484) and the polyclonal antibody to TLR3 was from Novus Biologicals (NBP2-24565). Polyclonal antibodies to NF-κB p65 and PKR were obtained from rabbit immunized with peptides, which are prepared by Genscript (Nanjing, China).

### Statistical analysis

The statistical analysis was performed with Student’s *t* test using GraphPad PRISM 6 software. *P* values less than 0.05 were considered statistically significant.
